# Patients don’t come with multiple choice options: essay-based assessment in UME

**DOI:** 10.1080/10872981.2019.1649959

**Published:** 2019-08-22

**Authors:** Jeffrey B. Bird, Doreen M. Olvet, Joanne M. Willey, Judith Brenner

**Affiliations:** a Department of Science Education, Donald and Barbara Zucker School of Medicine at Hofstra/Northwell, Hempstead, NY, USA; b Curricular Integration and Assessment, Zucker School of Medicine at Hofstra/Northwell, Hempstead, NY, USA

**Keywords:** Assessment, integrated curriculum, undergraduate medical education

## Abstract

Curricular revision efforts have resulted in learner-centered programs that value content integration and active learning. Yet, less attention has been placed on assessment methods that are learner-centered and promote assessment for learning. The use of context rich short answer question (CR-SAQ) exams in the preclinical years of medical school was evaluated to determine if this format aligns with the criteria for assessment for learning. Medical students and preclinical faculty members were sent a survey comprised of closed and open-ended questions about their experience using CR-SAQ exams. Data were analyzed using a mixed-method design. Open-ended responses were evaluated using thematic analysis within the framework of criteria for assessment *for* learning. A total of 274 students (94%) and 24 faculty (75%) completed the survey. Fifty four percent of students reported preferring a CR-SAQ exam format over multiple choice questions (MCQ) format. Quantitative data and qualitative comments by students supported that CR-SAQ exams aligned with criteria for assessment *for* learning, including acceptability, authenticity, educational effect, and the cueing effect. Student concerns included preparation for USMLE Step 1 exam, as well as the validity and reproducibility of CR-SAQ assessments. Faculty largely agreed with the benefits of the CR-SAQ, but were concerned about feasibility, acceptability and reproducibility. The CR-SAQ exam format assessment strategy supports assessment for learning in an undergraduate medical education setting. Both benefits and drawbacks of this method are presented, however students and faculty describe a broader impact that this assessment method has on their development as a physician.

## Introduction

Effective assessment serves multiple roles beyond the evaluation of the learner. The principle that ‘assessment drives learning,’ captures the capacity of assessment to stimulate learning, provide evidence that learners are making progress, measure the educational efficiency of a curriculum, inform instructors and administrators that programs are consistent with their missions, and, in medical education, to protect patients [1,2]. If assessment is to be successful, the assessment strategies must align with the educational goals [3]. The current era of curricular reform has seen a remarkable shift toward a more integrative, learner-centered approach to undergraduate medical education (UME) that promotes critical thinking and self-directed learning (SDL) [4]. Despite widespread introduction of fresh instructional approaches, there is little evidence that the UME community has similarly examined whether strategies used to assess medical knowledge in the preclinical years remain aligned with modern curricular goals. As revisions in curricula generate learner-centered programs that value content integration and active learning, it has been suggested that strategies that promote assessment *for* learning in addition to assessment *of*learning should be considered [5,6].

The medical education community has historically embraced the use of a multiple choice question (MCQ) format as assessment tools *of* learning [7] for several reasons, including feasibility, reproducibility and cost-effectiveness [1,7]. More recently, there has been a drive towards writing ‘context-rich’ MCQs in which test items are embedded in a clinical vignette [8]. Successfully answering questions embedded in patient scenarios requires more complex cognitive processes and problem-solving skills than answering more direct, factual knowledge questions [8].

While MCQs clearly have a place in an assessment toolbox, there is room for more. In reimagining assessment and in creating a tool that prioritizes assessment *for* learning, questions should be created to maximize the impact on the learner. Ideally, an assessment should motivate students to change their preparation by promoting critical thinking (educational effect, Table 1), [7] reduce the learner’s ability to rely on recognition (cueing effect) [9,10], improve later recall of content that has been assessed (generation effect) [11], and allow for meaningful feedback to further the learning cycle (catalytic effect) [2,7]. The assessment should also engage the learner in utilizing the same knowledge, skills, and attitudes needed in the workplace environment (authenticity) [12]. Some skills necessary for medical students to transition into their role as a physician include critical thinking, problem solving, and communication skills. Thus, ideally, an assessment should promote growth in these areas. Finally, students, as well as faculty, must buy into the assessment system for it to be considered valid (acceptability) [2].

**Table 1.** Criteria to be considered when developing as assessment strategy.

Criterion	Description	Reference
Construct validity	The assessment measures the intended knowledge or behavior	Gulikers 2004, Schuewirth 2004, Norcini 2011
Reproducibility	The assessment would yield the same results if repeated under similar circumstances	Norcini, 2011
Equivalence	The assessment yields same or similar scores when given across institutions	Norcini, 2011
Feasibility	The assessment is practical and realistic	Norcini, 2011
Authenticity	The assessment requires learners to apply the same knowledge, skills, and attitudes needed in real-life professional situations	Gulikers, 2004
Acceptability	All stakeholders (e.g., learners, instructors, administrators) agree that assessment process and results are credible	Norcini, 2011
Educational effect	The assessment prompts educationally beneficial preparation by learners	Norcini, 2011 Hift, 2014
Testing effect	Information retrieval improves later recall	Roediger 2011
• Generation effect	Assessment of content improves later recall	Taconnat, 2008 Roediger 2011
• Cueing effect	Answer recognition from a list of choices may overestimate student knowledge	Veloski 1999 Sam, 2018
Catalytic effect	Assessment results and feedback contributes to future learning	Norcini, 2011 Hift, 2014

It has been noted that open-ended format exams align with several of the effects of assessment *for* learning described above, including promoting deep learning strategies [9], emphasizing the need for recall rather than recognition, and providing a trace of learners’ thought process triggering opportunities for feedback [13]. Although open-ended questions are not frequently used in medical education, they may be an ideal mechanism to facilitate assessment *for* learning. Additionally, in the field of medicine, open-ended questions are authentic because physicians must be able to use divergent, critical thinking skills to recall and integrate basic and clinical science knowledge. As we know, patients do not come with multiple-choice options.

As a new medical school that matriculated its first class in 2011, the Donald and Barbara Zucker School of Medicine at Hofstra/Northwell (Zucker SOM) had the opportunity to build a brand-new educational program that aligned its pedagogical principles with curriculum delivery and assessment. We opted for an open-ended response exam format, which we term context rich short answer questions (CR-SAQ) to assess medical knowledge both formatively and summatively in all preclinical courses. Although the literature suggests open-ended formats are not superior to MCQ exams for assessing knowledge [7], we selected this format as one better aligned with the criteria critical in assessment *for* learning [2]. As more educators consider introducing essay-based exams into their assessment toolkits, this study aims to provide collateral information in the form of faculty and student perceptions. In order to evaluate this, we surveyed students and faculty on perceived benefits and drawbacks to a CR-SAQ assessment strategy.

## Methods

### Assessment strategy

At Zucker SOM, students participate in one integrated course at a time for six sequenced courses in the first two years. All grading is entirely pass/fail. Each integrated course has three curricular components (Mechanisms of Health, Disease and Intervention; Patient, Physician and Society; and Structure) for which application of students’ medical knowledge is assessed using CR-SAQ exams [14]. Exams are created as a collaborative effort amongst course directors for each of the three curricular components. Prior to exam creation, all faculty members participate in a one-time, sixty-minute faculty development exercise in which question and rubric writing are practiced. As new question-writers are identified, faculty development is scheduled. A typical exam includes eight to ten clinical scenarios, each with four to five associated short answer questions. Each year, course directors are expected to write at least ten new questions in order to continually expand the pool of questions available. CR-SAQ exams are completed on commercially available testing software (Examplify, Examsoft Worldwide, Inc., Dallas, TX) in a secure, proctored environment. Students are allowed four hours to complete the exam.

### Study design

**Figure 1.**
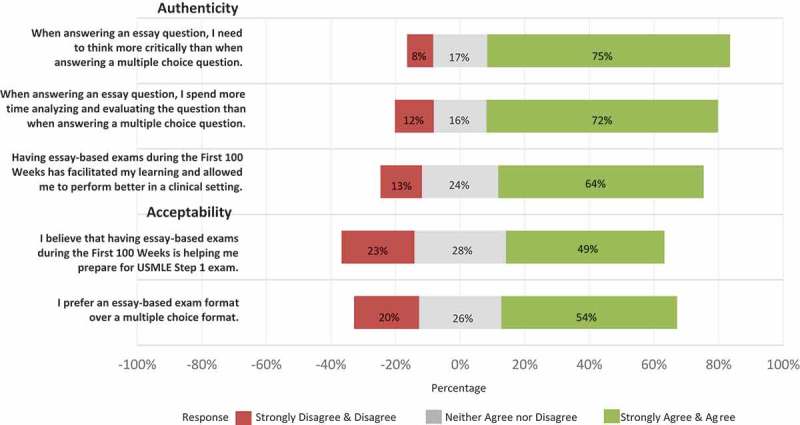
Student attitude regarding essay-based examinations (n = 274).

To gather student perspectives on the CR-SAQ strategy, survey questions were added to required course and clerkship evaluations in 2017. Data were collected from three cohorts of students corresponding to first year (MS1, N = 101), second year (MS2, N = 99), and third year (MS3, N = 91). At the time of the survey, MS1 students had completed 4 CR-SAQs, MS2 students had completed 11 CR-SAQs, and MS3 students had completed 13 CR-SAQs. In order to examine student’s perceptions of CR-SAQ exams, students were asked to rate the degree to which they agreed with the five statements, shown in Figure 1, using a 5-point Likert scale (Strongly Disagree, Disagree, Neither Agree nor Disagree, Agree, Strongly Agree). Two free response questions were also included: 1) What do you believe are the benefits of having an essay-based exam format; and 2) What do you believe are the drawbacks of having an essay-based exam format? To assess faculty attitudes towards our assessment strategy, a similar survey was sent to all Zucker SOM faculty members who graded an examination in 2015–2017 (N = 32).

### Data Analysis

Quantitative data were analyzed using SAS software, Version 9 (SAS Institute Inc., Cary, NC, USA). Descriptive statistics, including the frequency [N(%)] of responses for each of the 5-point Likert scale items, are provided. NQSR International Nvivo 11 software (QRS Technologies, Liverpool, NY) was used to analyze the open-ended comments. A framework analysis was used to guide the identification of individual codes [15,16]. Statements were coded based on the criteria to be considered when developing an assessment strategy (Table 1). Two raters (JW and DO) reviewed the written responses and applied codes to segments of data independently. The raters then met to discuss and agree on the final code book. The raters reviewed all codes applied to the comments to come to a consensus. This study was deemed exempt from review by the Hofstra University Institutional Review Board.

## Results

### Student perspective

A total of 274 students (94%) completed the survey. Results from the Likert-scale questions are shown in Figure 1. When addressing authenticity by asking students to draw upon their experience with MCQ exams and compare it to the assessment strategy used at Zucker SOM, 206 students (75%) agreed or strongly agreed that questions in our CR-SAQ format prompt more critical thinking, 197 students (72%) reported that CR-SAQs require more time to analyze than multiple choice, and 175 students (64%) reported that CR-SAQs facilitated learning and allowed students to perform better in a clinical setting. In addressing the criterion of acceptability, 149 students (54%) reported preferring a CR-SAQ exam format to an MCQ format, 70 (26%) had no preference, and 55 (20%) indicated they preferred an MCQ format.

**Figure 2.**
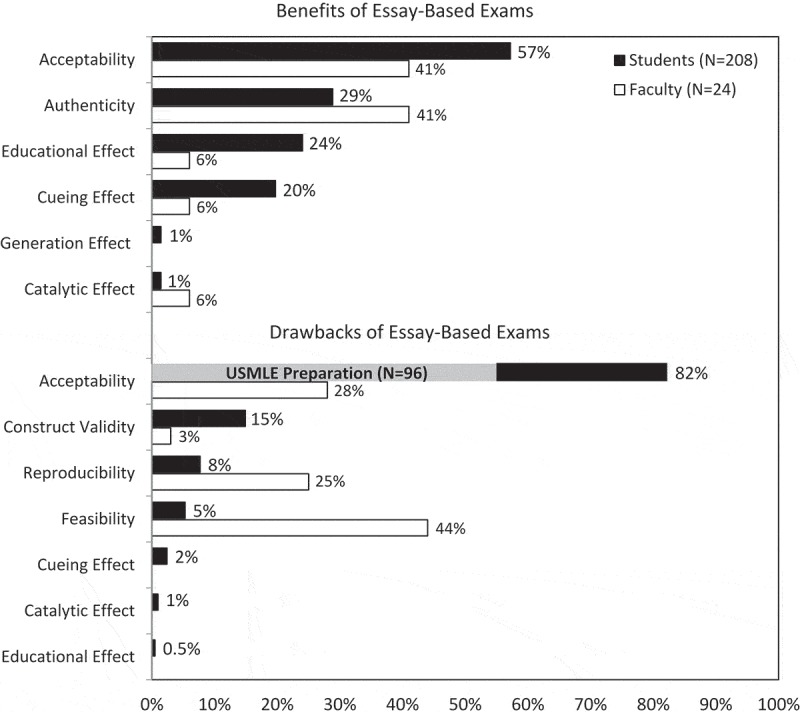
Percent of students and faculty whose open-ended comments were coded into one of the criteria for developing an educational assessment. Data for the coded comments describing the benefits (top) and drawbacks (bottom) of CR-SAQ exams are presented.

A total of 208 students provided responses to the free response questions. Overall, 513 segments of data were assigned codes, with 276 codes applied to the benefits of essay-based exams and 237 codes applied to the drawbacks. Percentages below represent the number of students whose responses were coded by a theme divided by 208 (the total number of students). Percentages are presented in Figure 2.

#### Benefits of essay-based exams

Table 2 shows representative comments by students on the benefits of CR-SAQ exams. One hundred and nineteen students (57%) identified the *acceptability* of CR-SAQs. Students appreciated the ability to show the grader what they knew about a given topic by explaining their reasoning, as well as the opportunity to earn partial credit.

**Table 2.** Representative comments on the benefits of having an essay-based exam form from students and faculty.

Theme	Student Comments	Faculty Comments
Acceptability	‘There is more wiggle room for partial credit and for dispelling away with the presupposition within multiple choice exams, that just because you may get a question wrong means you don’t understand the material. Essay-based exams allows for the exam grader to gauge the understanding and breadth of knowledge a really student holds.’	‘I think with an essay exam, faculty can determine with greater accuracy whether a student knows the information. That is to say, I don’t think a student can easily guess on an essay exam and get the answer right. It requires explanation in one’s own words, and separates those who understand a concept from those who don’t.’
Authenticity	‘The main benefit is that medicine in practicality does not function in multiple choice format- essay exams are preparing us to think critically as we move forward in our careers.’	‘It can also demonstrate students’ abilities to apply material to specific case-based scenarios, much as they would need to do in clinical settings.’
	‘ … essay exams, I believe, will better our communication skills and make us more critical and divergent thinkers in the clinical years.’	‘There is the ability to probe a deeper understanding of issues and to see how well the student can express that understanding. Patients do not present with a list of multiple choices.’
Educational Effect	‘In order to prepare I have to study a more dynamic picture of the material and have an understanding of the basic science deeper than that which I would need to take a multiple-choice test. This means that I am studying beyond the level of Step 1, therefore I am actually studying the things I need to know in order to be a good doctor.’	‘ … assessment drives learning, fosters a more connected/comprehensive approach to learning course material.’
	‘Even though essay questions are much harder to answer, it really promotes the studying of concepts over memorizing a bunch of facts.’	‘It causes the student to think deeply about the topic which can aid retention.’
Cueing Effect	‘It really forces us to not just recognize word-associations, but to actually understand processes that relate concepts and then being able to express/communicate that acquired knowledge, which requires more mental wherewithal/integration than just recognizing the letter or phrase that goes with the multiple-choice answer.’	‘I believe that an essay-based exam format challenges students to move beyond recognition of facts and to instead not only recall learned material but demonstrate an ability to connect information in a meaningful way in order to answer a question.’
Generation Effect	‘Being able to explain concepts and ideas make them stick easier in mind and allow for better recall.’	
Catalytic Effect	‘When reviewing my exam, I can see where my thought process went awry and try to fix that gap in my knowledge.’	‘Essay exams help me plan future learning sessions so I can be proactive to dispel unexpected misconceptions. When meeting with students to review exam performance, I have a much better idea of student areas of strengths and weaknesses.’

Sixty students (29%) wrote about the *authenticity* of essay-based exams by describing how they believe taking CR-SAQs will better prepare them for their role as a physician than MCQs. In particular, they perceive that CR-SAQs are important for developing clinical, communication, or critical thinking skills, as well as to foster divergent thinking and reasoning.

Fifty students (24%) described how they changed their study habits to prepare for the CR-SAQs. Consistent with the *educational effect*, students reported that they study harder, master the content and attain a deeper level of learning compared to how they would study for a MCQ exam.

Forty-one students (20%) noted that when taking MCQ exams, they rely on cueing. The *cueing effect* occurs when the correct answer can be identified using word or pattern recognition or can be identified through the process of elimination. CR-SAQs serve to prevent the cueing effect.

Both the *generation effect* and the *catalytic effect* are described as ways that assessment drives future learning. For each of these criteria, 3 students (1%) made comments in support of CR-SAQs that they will lead to improved recall later (generation effect), and that reviewing their exam responses at a later date is helpful in identifying where they went wrong (catalytic effect).

#### Drawbacks of CR-SAQ exams

Table 3 shows representative comments by students on the drawbacks of CR-SAQ exams. Although only 20% of students stated they would prefer an MCQ approach, 171 students (82%) expressed negative comments related to the criterion of *acceptability* of essay-based exams. Many of these students (N = 96, 56%) were concerned about being prepared to take the USMLE Step I exam, which uses an MCQ format. Another acceptability-related concern was the limited amount of material that could be tested with essay-based exams. Finally, students expressed that essay-based exams were harder and more stressful than multiple-choice exams.

**Table 3.** Representative comments on the drawbacks of having an essay-based exam form from students and faculty.

Theme	Student Comments	Faculty Comments
Acceptability	‘While I do not think there are “drawbacks” to essay based format, I think that they don’t help as much with the format of the step 1 exam. However I think that essay exams overall enhance learning, and learning should not be tailored to the format of the step one exam.’	‘This format is different from the USMLE exams that students will need to take, so it would be important to also make sure students feel adequately prepared for national standardized exams that are MCQ based.’
	‘Essay based exams take a much longer time to do, and can only assess a small amount of content that may not be reflective of the course.’	‘ … a multiple choice exam would be able to cover many more topics with more questions, because each question would take less time to answer and score. Also, it would better prepare the students for the type of exam they take for NBME.’
	‘The main drawback of an essay-based exam format is that it creates much higher stress for the students when studying.’	‘It is difficult to cover the range of information covered in the course.’
		‘I imagine this makes for a grueling testing experience for students.’
Construct Validity	‘Questions are often not completely precise and clear (in terms of what is being asked and what level of detail is needed in the answer), so that without the benefit of a selection of answer choices, this means that a lot of time is often spent just trying to figure out what the question is asking and what will constitute a satisfactory response.’	‘Students can come with answers that may be correct but not on the rubric.’
Reproducibility	‘Grading is a lot more subjective than with multiple choice which has one correct answer.’	‘Although we score based on a rubric, because the students are forming their own answers, it allows for a great deal of subjectivity in the scoring.’ ‘There can be a much wider range in depth and detail of responses, leading to increased challenges in creating a rubric to objectively assess responses.’
Feasibility	‘There is not enough time to give fully thought out answers that are actually explanatory for one’s thought process.’	‘From the faculty perspective, the grading can be onerous, not just from the sheer amount of time it requires, but essay-based exams also require more thought being put into the questions themselves and into the grading/rubric writing process. Totally worth it, though.’
Cueing Effect	‘The drawbacks to essay-based exams is that it makes the student have to own the material instead of being able to recognize topics based on multiple choice answers.’	

Thirty-one students (15%) expressed concern regarding the concept of *construct validity*, reporting that essay-based questions can have ambiguous wording or were open to interpretation. If students could not figure out what the question was asking, the intended knowledge could not be adequately measured.

*Reproducibility* was an issue raised by sixteen students (8%) who believed grading was subjective or unfair. If grading is subjective, then it is unlikely to be reproducible across various graders.

Eleven students (5%) mentioned that *feasibility* was an issue. Concerns were expressed when students stated that they could not complete the CR-SAQ in the amount of time provided. Feasibility concerns also arose when describing about the amount of time/effort required of both students and faculty.

Five students (2%) discussed the lack of a *cueing effect* with CR-SAQs as a drawback. These students preferred multiple choice questions because they could rely on recognizing the answer rather than recalling it.

### Faculty perspective

Faculty and students reported a very similar perspective of the CR-SAQ exam format. Of 32 faculty members surveyed, 24 responded (75% completion rate). Faculty acceptance of the CR-SAQ strategy mirrored that of students with slightly more than half (N = 13, 54%) preferring CR-SAQ to a multiple-choice format, while 25% disagreed with this view and another quarter of respondents remaining neutral (N = 5, 21%).

All faculty respondents provided responses to the free response questions. Overall, 66 segments of data were assigned codes, with 34 codes applied to the benefits of CR-SAQs and 32 codes applied to the drawbacks. Percentages below represent the number of faculty whose responses were coded by a theme divided by 24 (the total number of faculty who responded). Percentages are presented in Figure 2.

#### Benefits of CR-SAQ exams

Table 2 shows representative comments by faculty on the benefits of CR-SAQ exams. Fourteen faculty members (58%) identified the *acceptability* of CR-SAQs because they allow the grader to assess students’ depth of knowledge and provide partial credit.

Fourteen faculty members (58%) discussed the *authenticity* of CR-SAQs by preparing students for working in a clinical environment. In particular, they described skills, such as higher-order thinking, reasoning, and expression of knowledge, that are captured by this assessment format.

Some additional themes were represented by a small number of faculty comments. The *cueing effect* was described by two faculty (8%) in that CR-SAQs help eliminate guessing and challenge students to actively recall knowledge. Two faculty (8%) noted that this assessment format is helpful for providing feedback to students about their performance. They also reported that student answers helped them improve their own teaching by defining the content that students find most difficult (*catalytic effect*). Finally, two faculty (8%) noted that this form of assessment drives deeper learning (*educational effect*).

#### Drawbacks of CR-SAQ exams

Table 3 shows representative comments by faculty on the drawbacks of CR-SAQ exams. The main drawback expressed by faculty was the *feasibility* of CR-SAQs. Fourteen faculty (58%) expressed negative comments, including the amount of time and resources required to create questions, scoring rubrics, and grade exams.

Faculty also described concerns about *acceptability* of CR-SAQs (N = 9, 38%). Faculty noted that less material could be tested with CR-SAQs and that the USMLE Step I exam is in an MCQ format. One faculty member empathized with students that this method must be ‘grueling.’

*Reproducibility* was an issue raised by eight faculty (33%) who believed grading could be subjective. Finally, *construct validity* was raised as an issue by one faculty (4%) who explained that students might write an answer that is correct but not on the rubric.

## Discussion

In this study, we examined the use of a CR-SAQ assessment strategy at the Zucker SOM UME program. We sought to understand student and faculty perception of this assessment strategy using a framework that examines the ideal qualities of assessment *for* learning [7,8]. Overall, the study supports the use of CR-SAQ exams as having a role in UME assessment based on these criteria as demonstrated by the quantitative and qualitative responses. Both our faculty and students perceive that preparing to take a CR-SAQ exams promoted a deeper level of learning (*educational effect*). Next, many of our students and faculty believe that our CR-SAQ strategy better prepares them for their careers as clinicians (*authenticity*). In addition, students and faculty alike report using examination responses to diagnose learning and reasoning gaps (*catalytic effect*). Finally, in the area of *acceptability*, students cite both benefits as well as drawbacks. Students appreciate the notion of partial credit and the opportunity to demonstrate their knowledge to faculty, but both students and faculty voice concern about the ability of CR-SAQ to best prepare students for USMLE Step 1.

In considering the place that open-ended response exams could play in UME assessment, it is impossible to compete with the well-documented validity, reliability, and feasibility of the MCQ approach [17]. As such, this study does not attempt to argue against MCQs, but rather seeks to place open-ended questions on the ‘assessment table.’ We speculate that resistance to experiment with other assessment formats may be driven by two factors. First, the traditional role of testing has been assessment *of* learning and MCQ exams are an appropriate modality for this. Second, since MCQs remain the question type used in the USMLE Step examinations, it is natural to gravitate to MCQs in UME assessment. Indeed, when considering the drawbacks, our students and faculty overwhelmingly cite concern that CR-SAQs do not prepare students for the MCQ format of the USMLE Step 1 exam. Contrary to this perception, our institution’s cumulative pass rate for USMLE Step 1 is >99%, with our learners mean score consistently above the national mean. With the latest announcement that the National Board of Medical Examiners (NBME) will include several open-ended responses (short-answer questions) to the Medicine clinical science subject examination [18], we wonder if there will be a new drive in UME to include short answer questions in other versions of NBME exams.

Another major deterrent to implementation of an essay-based assessment strategy is the time and resources needed for faculty development and grading [19]. To this end, we have invested in considerable faculty development to address how best to write questions and rubrics. Once course directors have developed question banks, the biggest barrier is the time required to grade exams. To minimize the burden on course directors, we distribute grading responsibilities amongst a group of other faculty members. For example, six to eight faculty members typically grade a CR-SAQ exam with 30 questions. Our experience has been that a single question with 100 examinee responses requires two to three hours to grade. It has been suggested that as automated essay scoring systems become optimized, more medical schools may consider this assessment approach [20].

Despite safeguards built into our assessment strategy, the perception of subjective grading remains. To address this potential, we have put several checks in place throughout the grading process. First, we carefully review grading rubrics with all faculty graders and our system eliminates inter-rater variability by having single faculty members grade all answers for a given question. Next, by limiting the total number of points per question to a maximum of four, score assignment is narrowly defined on rubrics. Finally, all responses of examinations with potentially failing scores are carefully reviewed and discussed by a group of course directors prior to the assignment of a summative grade. Thus, in a pass/fail curriculum where students never see the numerical scores earned on individual assessments, the issue of subjectivity is mitigated, and the *catalytic effect* can prevail. Another concern by students and faculty is the observation that less content can be covered on an essay-based exam as compared to an MCQ test. While this can be viewed as a trade-off inherent to the nature of this assessment strategy, it is worth noting that students prepare to answer questions on all content; just because it was not assessed does not mean it was not learned.

Our study was unique for several reasons. First, although gaining traction, CR-SAQ exams are not commonly used in UME in the USA and little is written regarding their use in medical education. Second, there is a paucity of literature on assessment methods appropriate in modern UME curricula that stress integration and active learning pedagogies, which demand assessment *for* learning. In addition, we report both student and faculty perspectives and find that both sets of stakeholders find considerable value in this assessment modality while at the same time having similar reservations.

### Limitations and next steps

The generalizability of this study may be limited since it is based on a single school’s experience with a CR-SAQ assessment system. In addition, because the ZSOM has utilized CR-SAQ since its origination, the institution has not had experience overcoming cultural barriers. In order to broaden the perspective and promote generalizability, collaboration with other institutions utilizing similar modalities of assessment is a relevant next step. The current climate and reality of USMLE Step 1 also makes it important to investigate Step 1 scores in other schools utilizing an essay-based approach to assessment. In addition to perception data, psychometric data (reliability, feasibility) may be useful as other schools begin to consider the potential for learning from this form of assessment. Lastly, validation studies between MCQ vs. essay-based exam assessment strategies should be conducted.

### Conclusions

From the curricular perspective, we conclude that CR-SAQ exams offer an appropriate platform to assess our learners, as this modality is most congruent with all other aspects of our program. CR-SAQ exams enable both the learner and instructor to view a trace of the thought process that led to an answer. This can be particularly powerful for the learner who is struggling and for the instructor in identifying student misconceptions. We believe that a key to the success of this assessment strategy is the culture we have sown – one that includes buy-in from faculty who recognize the value of essay-based assessments and of students who recognize that assessment based on integrative, creative, critical thinking will prepare them for clinical practice.

## Data Availability

Raw data were generated at the Zucker School of Medicine. Derived data supporting the findings of this study are available from the corresponding author (JB) on request.
